# LATE-NC staging in routine neuropathologic diagnosis: an update

**DOI:** 10.1007/s00401-022-02524-2

**Published:** 2022-12-13

**Authors:** Peter T. Nelson, Edward B. Lee, Matthew D. Cykowski, Irina Alafuzoff, Konstantinos Arfanakis, Johannes Attems, Carol Brayne, Maria M. Corrada, Brittany N. Dugger, Margaret E. Flanagan, Bernardino Ghetti, Lea T. Grinberg, Murray Grossman, Michel J. Grothe, Glenda M. Halliday, Masato Hasegawa, Suvi R. K. Hokkanen, Sally Hunter, Kurt Jellinger, Claudia H. Kawas, C. Dirk Keene, Naomi Kouri, Gabor G. Kovacs, James B. Leverenz, Caitlin S. Latimer, Ian R. Mackenzie, Qinwen Mao, Kirsty E. McAleese, Richard Merrick, Thomas J. Montine, Melissa E. Murray, Liisa Myllykangas, Sukriti Nag, Janna H. Neltner, Kathy L. Newell, Robert A. Rissman, Yuko Saito, S. Ahmad Sajjadi, Katherine E. Schwetye, Andrew F. Teich, Dietmar R. Thal, Sandra O. Tomé, Juan C. Troncoso, Shih-Hsiu J. Wang, Charles L. White, Thomas Wisniewski, Hyun-Sik Yang, Julie A. Schneider, Dennis W. Dickson, Manuela Neumann

**Affiliations:** 1 University of Kentucky, Rm 575 Todd Building, Lexington, KY USA; 2 University of Pennsylvania, Philadelphia, PA USA; 3 Houston Methodist Hospital, Houston, TX USA; 4 Uppsala University, Uppsala, Sweden; 5 Rush University Medical Center, Chicago, IL USA; 6 Illinois Institute of Technology, Chicago, IL USA; 7 Newcastle University, Newcastle Upon Tyne, UK; 8 University of Cambridge, Cambridge, UK; 9 University of California, Irvine, CA USA; 10 University of California, Davis, CA USA; 11 Northwestern University Medical Center, Chicago, IL USA; 12 Indiana University, Indianapolis, IN USA; 13 University of California, San Francisco, CA USA; 14 Unidad de Trastornos del Movimiento, Servicio de Neurología Y Neurofisiología Clínica, Instituto de Biomedicina de Sevilla, Hospital Universitario Virgen del Rocío/CSIC/Universidad de Sevilla, Seville, Spain; 15 University of Sydney, Sydney, NSW Australia; 16 Tokyo Metropolitan Institute of Medical Science, Tokyo, Japan; 17 Institute of Clinical Neurobiology, Vienna, Austria; 18 University of Washington, Seattle, WA USA; 19 Mayo Clinic, Jacksonville, FL USA; 20 Tanz Centre for Research in Neurodegenerative Disease, University of Toronto, Toronto, Canada; 21 Department of Laboratory Medicine and Pathobiology, University of Toronto, Toronto, Canada; 22 Laboratory Medicine Program, University Health Network, Toronto, Canada; 23 Institute of Neurology, Medical University of Vienna, Vienna, Austria; 24 The Cleveland Clinic, Cleveland, OH USA; 25 The University of British Columbia, Vancouver, BC Canada; 26 University of Utah, Salt Lake City, UT USA; 27 Stanford University, Stanford, CA USA; 28 University of Helsinki and Helsinki University Hospital, Helsinki, Finland; 29 University of California San Diego, San Diego, CA USA; 30 Tokyo Metropolitan Geriatric Hospital & Institute of Gerontology, Tokyo, Japan; 31 Washington University, St. Louis, MO USA; 32 Columbia University, New York, NY USA; 33 Laboratory for Neuropathology, Department of Imaging and Pathoogy, and Leuven Brain Institute, KU Leuven, Leuven, Belgium; 34 Department of Pathology, University Hospital Leuven, Leuven, Belgium; 35 Johns Hopkins University, Baltimore, MD USA; 36 Duke University, Durham, NC USA; 37 University of Texas Southwestern Medical Center, Dallas, TX USA; 38 New York University Grossman School of Medicine, New York, NY USA; 39 Department of Neurology, Brigham and Women’s Hospital, Harvard Medical School, BostonBoston, MAMA USA; 40 University of Tübingen and DZNE Tübingen, Tübingen, Germany

**Keywords:** Dementia, Processes, NCI, TDP-43, FTD, Stages, Hippocampal sclerosis, Neuroanatomy, Aging

## Abstract

An international consensus report in 2019 recommended a classification system for limbic-predominant age-related TDP-43 encephalopathy neuropathologic changes (LATE-NC). The suggested neuropathologic staging system and nomenclature have proven useful for autopsy practice and dementia research. However, some issues remain unresolved, such as cases with unusual features that do not fit with current diagnostic categories. The goal of this report is to update the neuropathologic criteria for the diagnosis and staging of LATE-NC, based primarily on published data. We provide practical suggestions about how to integrate available genetic information and comorbid pathologies [e.g., Alzheimer’s disease neuropathologic changes (ADNC) and Lewy body disease]. We also describe recent research findings that have enabled more precise guidance on how to differentiate LATE-NC from other subtypes of TDP-43 pathology [e.g., frontotemporal lobar degeneration (FTLD) and amyotrophic lateral sclerosis (ALS)], and how to render diagnoses in unusual situations in which TDP-43 pathology does not follow the staging scheme proposed in 2019. Specific recommendations are also made on when not to apply this diagnostic term based on current knowledge. Neuroanatomical regions of interest in LATE-NC are described in detail and the implications for TDP-43 immunohistochemical results are specified more precisely. We also highlight questions that remain unresolved and areas needing additional study. In summary, the current work lays out a number of recommendations to improve the precision of LATE-NC staging based on published reports and diagnostic experience.

**Supplementary Information:**

The online version contains supplementary material available at 10.1007/s00401-022-02524-2.

## Introduction

Transactive response DNA-binding protein of 43 kDa (TDP-43) pathology is prevalent in aging brains and is often associated with cognitive impairment or dementia [114]. Age-related TDP-43 pathology and associated clinical features have been described by many investigators over past decades [6, 14, 28, 30, 31, 46, 49, 59, 66, 70, 72, 85, 103, 106, 117, 125, 137, 145, 146, 148, 155, 158, 161], but a consensus nomenclature was lacking until recently. In 2019, a multidisciplinary consensus group suggested terminology for age-related TDP-43 pathologic changes associated with cognitive impairment. The disease was designated “limbic-predominant age-related TDP-43 encephalopathy” (LATE) [114], and guidelines were suggested for post-mortem evaluation and staging of LATE neuropathologic changes (LATE-NC). This terminology has been adopted widely [16, 25, 48, 50–52, 57, 74, 81, 95, 100, 132, 135, 138, 153]. However, diagnostic ambiguities and criticisms of the staging scheme have emerged [22, 61, 115].

At least four shortcomings have been identified in the 2019 LATE-NC guidelines: (1) anatomic regions for sampling were recommended, but the implications of TDP-43 immunopositivity in subregions were not precisely defined; (2) it was not clear how additional information on genetic findings and other pathologies should be incorporated into reports of LATE-NC; (3) there was minimal guidance on how to separate LATE-NC from other TDP-43 proteinopathies; and (4) some cases with TDP-43 pathology in aging could not be readily classified into LATE-NC stages.

The aims of the present paper are to remedy these shortcomings, to provide data to clarify the proper use of the LATE-NC staging system, and to indicate where the diagnosis of LATE-NC may not be appropriate. The overarching goal is to provide additional precision about LATE-NC diagnosis for neuropathologists. These goals are important, since brain autopsy remains the gold-standard for neurodegenerative disease diagnoses. New recommendations and clarifications are proposed for the LATE-NC staging system, guided by published data, findings reported at the LATE 2022 Conference [1] and diagnostic experience.

## LATE-NC: recommendations for anatomic sampling and staining

**Fig. 1:**
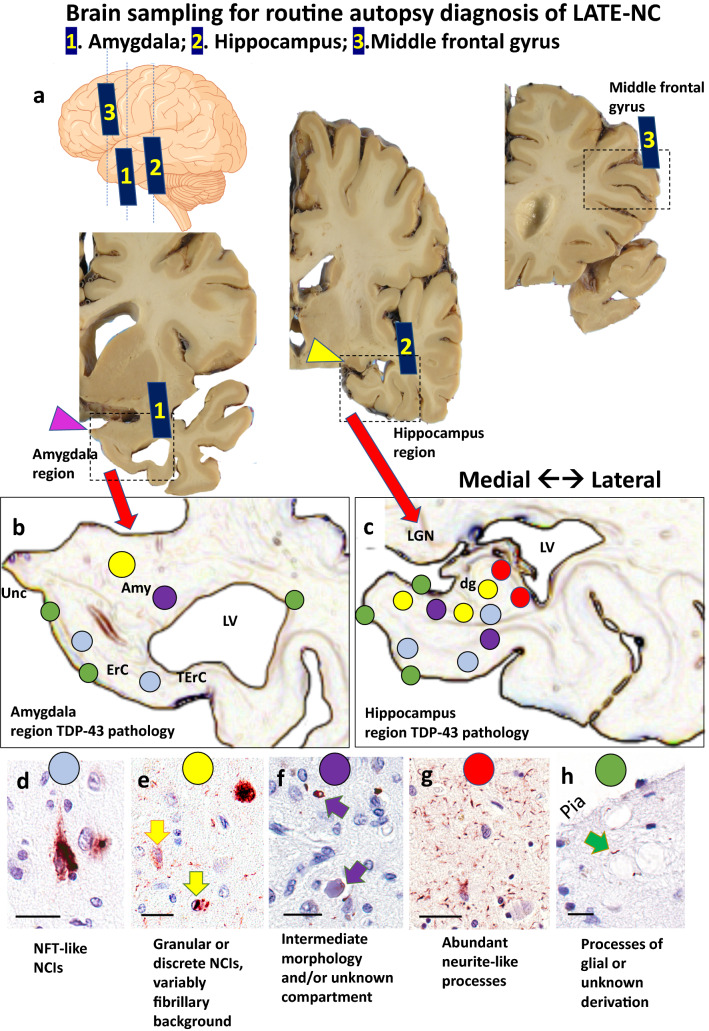
Anatomical regions of interest for tissue sampling and typical findings in routine autopsy diagnosis of LATE-NC. At autopsy, tissue portions for sampling include amygdala, mid-level hippocampus, and middle frontal gyrus. The levels of sections are shown in the cartoon form (upper left) with gross photographs of hemi-brains cut in the coronal plane (Panel a). Note that the amygdala is preferably sampled for TDP-43 immunohistochemical staining at the level of the uncus (pink arrowhead, Sect. 1), the hippocampus at the level of the lateral geniculate nucleus (yellow arrowhead, Sect. 2), and the middle frontal gyrus (Sect. 3) is sampled rather than other portions of frontal cortex. Panels b and c are representations of the amygdala region and hippocampal region, showing both the local anatomy and a cursory depiction of the subtypes of TDP-43 pathology that are generally found in those regions with corresponding colored circles in panels d-h. Panel d shows a neuronal TDP-43 + inclusion reminiscent of a neurofibrillary tangle. Panel e depicts a different TDP-43 pathologic appearance with a granular NCI (arrow with red outline), neuronal intranuclear inclusion (arrow with blue outline), and TDP-43 + fibrillary material in the background. This pattern is reminiscent of FTLD-TDP type A. TDP-43 pathology can also be present around vascular components such as capillaries (termed Lin Bodies after Ref. [87]) or in unknown histologic compartments as shown in Panel f. In some regions, the predominant TDP-43 + pathology is fine non-tapering neurite-like processes (Panel g). A different type of TDP-43-immunoreactive cell processes can be seen in the sub-pial region, often near corpora amylacea (arrow in Panel h). Scale bars = 50 microns (d); 30 microns (e); 30 microns (f); 100 microns (g); and, 30 microns (h). Abbreviations: Amyg: amygdala proper; dg: dentate granule layer of hippocampus; ErC: entorhinal cortex; LGN: lateral geniculate nucleus; LV: lateral ventricle; NCI: TDP-43 immunoreactive neuronal cytoplasmic inclusions; NFT: neurofibrillary tangles; TErC: transentorhinal cortex; Unc: uncus

Regarding brain tissue collected at autopsy to assess LATE-NC, this update does not propose additional sampling relative to the 2019 consensus recommendations [114]. However, there is a need to clarify the implications of the immunostaining results within specific regions of interest. Three anatomical regions are recommended for sampling, with the following suggestions (Fig. 1):

1. Amygdala region: this refers to the amygdala and surrounding structures at the level of the uncus, including adjacent entorhinal [Brodmann area (BA) 28], transentorhinal (BA35), anterior temporal (BA36) cortices and anterior parts of the hippocampus and the subiculum/presubiculum, sub-pial, subependymal regions, and white matter. The pathology is scored as positive in this region if aberrant TDP-43 immunoreactivity is seen anywhere on the section that contains amygdala and uncus (not just within amygdala proper).
2. Hippocampus region: this refers to the hippocampus and associated medial temporal cortical structures at the level of the lateral geniculate nucleus. Areas of interest may include fornix, sub-pial region, periventricular region, dentate granule cells, mid-level temporal cortex (BA36), and white matter. This region is considered to be positive if any part of the section has TDP-43 immunoreactive pathology.
3. Middle frontal gyrus (corresponding roughly to BA46). TDP-43 immunoreactive cytoplasmic inclusions in any part of this section is considered to be positive.

Supplemental File 1 (online resource) provides additional detail on the specific anatomical regions and subregions of interest, as pertains to LATE-NC staging.

The LATE-NC staging guidelines suggested that TDP-43 immunohistochemistry should be performed as part of the neuropathologic evaluation of all older individuals’ brains [114], but there was no specific recommendation on the staining methods used. The antibody used for pathologic diagnosis and associated protocols may affect the ability to detect TDP-43 inclusions [40, 53, 79]. Phosphorylation-specific antibodies, with those against pS409/410 being the most commonly used [44, 120], robustly label pathologic TDP-43 inclusions and do not stain normal (nonphosphorylated) nuclear TDP-43 protein, thereby facilitating identification of aggregates [35] but precluding the visualization of the loss of normal nuclear TDP-43 protein in inclusion bearing cells and nuclear staining as internal positive control. Antibodies that are phosphorylation independent allow for labeling of inclusions (albeit slightly less sensitively), while also enabling assessment of normal nuclear TDP-43 protein [40, 53, 79]. In an informal survey of U.S. Alzheimer’s Disease Research Centers, approximately 2/3^rd^ of the neuropathologists depended on antibodies against the phosphorylated Ser409/Ser410 TDP-43 epitope [71]. More standardization may be achievable in the future, but there is still not a current consensus on a prescribed set of reagents for TDP-43 detection.

## LATE-NC: recommendations for pathological staging

LATE-NC staging, like other neurodegenerative disease staging systems [12, 18–20, 140], is based on anatomical regions affected by the neuropathologic changes. Following prior studies which incorporated analyses of TDP-43 immunohistochemical data from more anatomical regions [63, 64, 105, 107, 146, 162], the 2019 consensus guidelines for LATE-NC suggested that TDP-43 pathology progressed in a stereotypical pattern: LATE-NC Stage 1 corresponds to TDP-43 pathology in the amygdala, Stage 2 corresponds to TDP-43 pathology in the amygdala and hippocampus, and Stage 3 corresponds to TDP-43 pathology in the amygdala, hippocampus, and middle frontal gyrus [114]. If other neocortical regions (orbitofrontal cortex, or temporal neocortex) are stained and show neuronal cytoplasmic inclusions (NCIs), but NCIs are not seen in the middle frontal gyrus, this would not represent LATE-NC Stage 3. Some such LATE-NC Stage 2 cases would be expected, because orbitofrontal cortex and temporal neocortex (as well as some other regions) are affected earlier than middle frontal gyrus in the anatomical progression of LATE-NC [64, 105]. While most cases can be readily assigned to a given LATE-NC stage using those criteria, recent reports have described cases that depart from the staging scheme for one or more reasons [21, 29, 41, 62, 143, 147]. Below are described strategies to address various diagnostic challenges in a data-driven and standardized manner.

### *Brains with TDP-43* + *NCI detected in the hippocampal region, but not in the amygdala region*

In recently published studies from five separate autopsy series [29, 41, 80, 109, 143], a small subset of cases from each cohort was reported with TDP-43 pathology in the hippocampal formation, but not in the amygdala. This pattern is not accounted for in the original LATE-NC staging guidelines [114]. LATE-NC cases with hippocampal TDP-43 + NCIs, but none in amygdala region, are unusual (< 2% of LATE-NC cases), and the TDP-43 pathology usually involves the anterior hippocampus. These cases lacking detectable amygdala TDP-43 pathology may be due in part to sampling issues, and the clinical implications are unclear. More work is required to confirm that hippocampus-only TDP-43 pathology is appropriately diagnosed as LATE-NC. At present, because such cases have a low burden of TDP-43 pathology overall, and we do not know of an example of an individual with cognitive impairment lacking other pathologies, we recommend that this pattern of TDP-43 pathology be designated LATE-NC Stage 1.

To account for the varied pathologic patterns in LATE-NC Stage 1 cases, a system for differentiating subtypes of LATE-NC Stage 1 cases is now recommended. This classification may be most suitable for academic research centers, and should be considered optional for diagnosticians. In this system, a “conventional” case with TDP-43 immunoreactive NCI(s) in amygdala but not hippocampus would be diagnosed as LATE-NC Stage 1a, whereas an unusual case with NCI(s) in hippocampus but none detected in the amygdala region would be diagnosed as LATE-NC Stage 1b (Table 1).

**Table 1:**
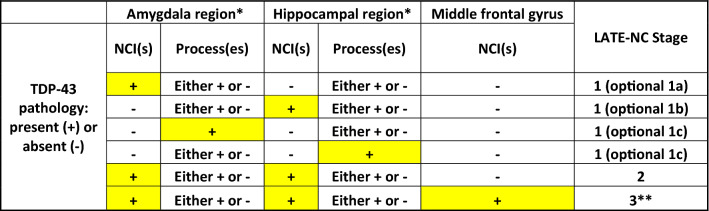
Specific pathological combinations and corresponding recommendations for LATE-NC staging

^*^Amygdala region and hippocampal region refer to anatomical areas on the same slide

^**^See recommendations to distinguish LATE-NC Stage 3 from FTLD-TDP and ALS

### *Brains with TDP-43* + *cell processes, but no TDP-43* + *neuronal cytoplasmic inclusions (NCI)*

In LATE-NC, TDP-43 proteinopathy is often detected in cellular compartments outside of the neuronal cell body [11, 29, 39, 116]. A recent study focusing on cases with minimal or incipient LATE-NC described cases without NCI, but with short, non-branching TDP-43 immunoreactive structures that frequently localized around corpora amylacea, and/or in aging-related tau astrogliopathy (ARTAG) pathology [29, 37, 110, 128]. Because of the apparent astroglial derivation of some TDP-43 pathology outside of neuronal cell bodies, we use the term “processes”, as opposed to “neurites”, for these common TDP-43-immunoreactive structures. TDP-43-positive processes were detected in the sub-pial zone overlying the corticomedial region of the amygdala and/or in the periventricular white matter subjacent to amygdala. This pattern was common—aberrant TDP-43 was found only in cellular processes in approximately one-third of LATE-NC cases [29]. Another recent study confirmed that there are cases with TDP-43 + processes but lacking TDP-43 + NCIs [80].

We propose that cases with only TDP-43-positive processes, and no NCI in the amgydala and hippocampus regions, should be diagnosed as LATE-NC Stage 1. It is emphasized that LATE-NC Stage 2 requires at least a single NCI to be present in each of both the amygdala and hippocampal regions. In the context of the optional LATE-NC Stage 1 subtyping system as described above, any case with TDP-43 immunoreactive processes only (no NCI) would be LATE-NC Stage 1c, even if the TDP-43 + processes are in the hippocampus region. The various possible neuropathologic changes seen in LATE-NC Stage 1, and their relevant diagnoses, are shown in detail in Table 1. This staging scheme will help clarify diagnostic practice, but we also recognize that more work is required to understand the implications of aberrant TDP-43 protein in processes but not NCIs.

### *Brains with comorbid Alzheimer’s disease neuropathologic changes (ADNC), Lewy bodies, and/or granulovacuolar degeneration*

It has been recognized for over a decade that TDP-43 pathology is often comorbid with ADNC, Lewy body pathology, or both [8, 10, 46, 66, 67]. However, these pathologic features also are commonly seen—even when quite severe—in the absence of each other [51, 52, 88, 113, 147, 153, 154], so they are at least partly independent. Many studies have demonstrated that LATE-NC is associated with additive cognitive impairment for a given amount of other pathologic changes [4, 16, 17, 72, 103, 111, 127]. Therefore, we recommend that descriptions of the individual diseases (and their neuropathologic stages) be rendered as separate line diagnoses on autopsy reports. A potential pitfall for the assessment of LATE-NC is the fact that some phospho-TDP-43 antibodies also detect granulovacuolar degeneration (GVD) [51, 69, 76, 139, 151]—this staining is not considered to represent LATE-NC. For more data and discussion related to immunostaining of GVD, see Refs [51, 69, 76, 77, 141, 151].

## Differentiating LATE-NC from other pathologies

### *Differentiating LATE-NC Stage 3 from FTLD-TDP*

TDP-43 was initially identified as a protein that forms abnormal intracellular aggregates in the most common pathology associated with clinical frontotemporal dementia (FTD) syndromes, now termed frontotemporal lobar degeneration with TDP-43 pathology (FTLD-TDP) and in ALS [122]. The defining feature of FTLD-TDP is the presence of NCIs and dystrophic TDP-43 immunoreactive processes in affected frontotemporal cortices [23, 93, 94, 122]. Differences in the morphologic features, abundance, and laminar distribution of TDP-43 pathology in the cerebral neocortex allow for recognition of at least five subtypes of FTLD-TDP, each with relatively specific clinical and genetic correlations and increasingly recognized molecular differences in the nature of the TDP-43 aggregates [84, 90, 92, 119, 121, 124]. In addition, each FTLD-TDP subtype has a fairly distinct pattern of limbic and subcortical involvement that is helpful in their classification [65, 91]. Nevertheless, potential pitfalls in the FTLD-TDP subtyping have been discussed due to the presence of mixed subtypes (usually FTLD-TDP types A + B), and overall low inter-rater agreement in separating FTLD-TDP types A and B [5, 9, 90].

Cases with TDP-43 pathology mostly restricted to the medial temporal lobes (LATE-NC Stages 1 or 2) do not fulfill criteria for FTLD-TDP and should be classified as LATE-NC. By contrast, boundaries between LATE-NC Stage 3 and FTLD-TDP are more challenging (~ 11% of LATE-NC cases in community-based cohorts were Stage 3 [113]). Ancillary measures, such as neuronal loss and gliosis, as well as superficial laminar spongiosis, involvement of further cortices and subcortical nuclei and white matter in FTLD-TDP, and the morphology of inclusions may help differentiate genuine FTLD-TDP from LATE-NC Stage 3. More work is needed to delineate aspects of biological overlap and differences between LATE-NC and FTLD-TDP, particularly for FTLD-TDP in advanced old age [22].

Based on current knowledge, our recommendations are as follows:

- TDP-43 pathology in LATE-NC has histomorphologic features that may be similar to FTLD-TDP type A or less commonly type B [8, 114, 131, 149]. By contrast, all cases with FTLD-TDP types C, D, or E patterns [84] should be classified as FTLD-TDP and not LATE-NC.
- The severity of TDP-43 pathology in the middle frontal gyrus has been described as a useful indicator to differentiate LATE-NC Stage 3 from FTLD-TDP [131]. Specifically, more than 15 TDP-43 immunoreactive inclusions (NCIs and/or threads) per high power (40 × objective) microscopic field in the middle frontal gyrus had a high sensitivity and specificity in differentiating FTLD-TDP from LATE-NC [131]. More than 15 TDP-43-immunoreactive pathological structures per 40 × microscopic field in the middle frontal gyrus favor a diagnosis of FTLD-TDP or a descriptive diagnosis of the pathologies observed, rather than LATE-NC.
- In cases with substantial TDP-43 pathology in the middle frontal gyrus, it is recommended to expand regions sampled for TDP-43 pathology and to include regions more typically affected in FTLD-TDP, such as subcortical structures, including basal ganglia, medulla (hypoglossal nucleus), and spinal cord. TDP-43 pathology in these regions supports a diagnosis of FTLD-TDP or ALS.
- Cases with known pathogenetic mutations in FTLD-TDP-related genes (e.g., *GRN*, *C9orf72*, *VCP,* and others [99, 150]) should not be classified as LATE-NC. Notably, to identify *C9orf72* repeat expansion carriers, immunohistochemistry of cerebellum with antibodies against p62 or dipeptide repeat proteins can be used as surrogate markers. This method is recommended in standard histopathologic screening if genetic analyses are not available [89].
- Cases with comorbid FTLD-TDP and LATE-NC may exist, but there currently is no consensus about how to definitively diagnose such cases as distinct from FTLD-TDP alone.

### *Differentiating LATE-NC from amyotrophic lateral sclerosis (ALS)*

Another important disease associated with TDP-43 proteinopathy is ALS. A four-tiered staging system of TDP-43 neuropathologic changes in ALS has been described by Brettschneider et al. [20] based on an autopsy cohort of ALS patients with average age at death of 63 years. TDP-43 pathology in the hippocampus region was designated stage 4 and was found in ~ 30% of cases. While, in this study, no significant differences regarding age at disease onset, or age at death, were observed between different stages of TDP-43 pathology, a recent study found medial temporal lobe TDP-43 pathology in 8/8 older ALS cases (> 75 years of age at death) [101], suggesting that in ALS, there may be pathology overlapping with LATE-NC. Yet, the TDP-43 neuropathologic changes in ALS differ from LATE-NC: motor neuron TDP-43 pathology has not been reported in LATE-NC, but is an early site of TDP-43 pathology in ALS [20], and is even seen in some FTLD-TDP cases without clinical ALS [32]. Until methods are available to distinguish TDP-43 pathology associated with LATE-NC from limbic pathology in ALS, we recommend avoiding the term LATE-NC in the context of sporadic and familial ALS.

### *Brains with unusual TDP-43 pathologies*

Research continues to find expanding implications of aberrant TDP-43 protein in human diseases. TDP-43 pathology is now known to occur in more than 20 different conditions spanning neurodegenerative, developmental, trauma-related, myopathic, and even neoplastic disease categories [2, 13, 26, 27, 78, 83, 129, 133, 134, 157]. For example, TDP-43 pathology is often seen in corticobasal degeneration, Perry syndrome, Alexander disease, and the Parkinsonism–dementia complex diseases of Guam and Kii [43, 75, 148, 152, 156]. However, the morphology and anatomical patterns of the TDP-43 pathology in those conditions often differ from that in LATE-NC, so the diagnosis of LATE-NC should be avoided in these conditions. For persons with a clinical history of brain trauma—traumatic brain injury (TBI) and/or chronic traumatic encephalopathy (CTE)–TDP-43 pathologic changes may be seen, but not necessarily related to LATE-NC [3, 60, 96, 97, 126]. More work is required to develop a data-driven consensus of best practices for diagnosing brain trauma-related TDP-43 pathology.

## Guidance in autopsy reports

Brain autopsy reports of individuals across the spectrum of dementias convey complex information reflecting the (often multifactorial) nature of the underlying diseases. That complexity, along with technical nomenclatures, can make it difficult, especially for patients’ families and loved ones, to understand the implications of TDP-43 pathology. It may be helpful for practicing neuropathologists to provide interpretive summaries, but we emphasize that this should be a separate comment with a focus on interpretation of findings in nontechnical language [82].

What are the implications of LATE-NC staging results? Clinical–pathological studies demonstrated that LATE-NC is associated with cognitive impairment (often with amnestic features), independent of other disease processes [17, 36, 42, 51, 58, 62, 68, 72, 88, 103, 104, 111, 130, 132, 135]. For example, in an attributable risk analysis based on all observed pathologic changes that may contribute to cognitive impairment in a large community cohort, LATE-NC accounted for more than 15% of identified amnestic dementia risk [16, 114]. Similar to other age-related neurodegenerative disease processes, it is not possible to confidently predict clinical implications of LATE-NC in a given individual, especially if there are multiple concurrent pathologies [24, 98, 108, 109, 112, 143, 146].

The following are possible text templates to complement the diagnostic findings in cases with LATE-NC:

- LATE-NC Stage 1 indicates the early/incipient stage of LATE-NC, analogous to early stages of other brain and systemic diseases where pathologies are present before outward clinical signs/symptoms (e.g., Braak NFT Stages I–II in Alzheimer’s disease Neuropathologic Changes). The presence of LATE-NC Stage 1 may be compatible with normal cognition or may have only a relatively small additive impact on cognitive function.
- LATE-NC Stage 2 indicates that TDP-43 neuronal cytoplasmic inclusions are detected more broadly in the brain. LATE-NC Stage 2 is usually associated with some impairment of memory or global cognition or both [114], with more cognitive impairment in cases with comorbid hippocampal sclerosis.
- LATE-NC Stage 3 indicates the most advanced stage of LATE-NC in terms of neuroanatomic distribution of TDP-43 pathologic burden in the brain. LATE-NC Stage 3 is usually associated with some degree of impairment of memory or global cognitive impairment [114], and with more cognitive impairment in cases with comorbid hippocampal sclerosis.

## Future directions and knowledge gaps related to LATE-NC

Considering the major public health impact of LATE-NC, there are many aspects of this disorder that merit further research. Of greatest urgency is the need for a sensitive and specific method to diagnose LATE during life, ideally at early stages of disease, to enable focused recruitment into clinical trials. The involvement of aberrant TDP-43 in a wider range of brain areas, and the role(s) of astrocytes in pathogenesis could also be foci of further scholarship. Systematic neuropathological and clinicopathological studies will inform if the staging system needs modifications to include intermediate stages. Additional pressing research is needed to define and stratify risk factors and interactions with other diseases, including various neurodegenerative and neurovascular disorders (see, for example, Refs [15, 37, 128]). Observations in more diverse communities are critical, since socioeconomic, cultural, and/or ancestral aspects may influence clinical–pathological correlation [55, 109, 123, 146]. (Some recent scholarship has evaluated LATE-NC in cohorts other than Whites [104, 109, 123, 146].)

It may be possible to discover molecular subtypes of LATE-NC using immunophenotyping, transcriptome profiling, and genotyping or other approaches, which has potential implications for prognosis and therapy. Indeed, there has already been significant progress along these lines [119, 143]. In particular, pathologic heterogeneity in LATE-NC has been demonstrated particularly in the early phases of LATE-NC, which occurs in different patterns and appears to originate at one of several different anatomic locations in the amygdala region [29, 62]. Certain parameters and features (e.g., comorbid Lewy body disease) have been associated with differing patterns of LATE-NC [29, 62]. A genetic risk factor for LATE-NC is *TMEM106B* [33]. Notably, the *TMEM106B* risk allele frequency was elevated in all identified LATE-NC patterns, and the different TDP-43 pathologic patterns observed in cases with mild pathology tended to converge in more severely affected brains [29]. We conclude that cases meeting criteria for LATE-NC encompass both heterogeneity and meaningful commonalities, but there currently is no agreement on means to differentiate LATE-NC subtypes.

Another area of uncertainty is the relation of LATE-NC to hippocampal sclerosis (HS). HS in the elderly is strongly associated with LATE-NC [7], with 75–90% of HS cases in aging being seen in cases with LATE-NC [34, 38, 47, 103, 106, 118]. The presence or absence of comorbid HS pathology does not affect LATE-NC staging. Further, HS is a diagnostic term that refers to distinctly different disease processes. For example, the diagnosis of HS is commonly applied in the context of persons with epilepsy, where the pathogenesis is very different (and lacks TDP-43 proteinopathy) in comparison to LATE-NC [118, 136, 142]. At autopsy, sensitive detection of HS requires bilateral sampling, because HS in LATE-NC is often unilateral [118, 161]. In hippocampi affected by severe HS, pyramidal cell dropout is extensive, and hippocampal atrophy can be extreme [6]; however, in some individuals with LATE-NC, cell loss is segmental (neuronal loss in some portions of the hippocampus and/or subiculum, but not others) [54]. These factors increase the likelihood of poor inter-rater agreement on the neuropathologic diagnosis of HS, and help explain the differing frequencies of HS reported in various autopsy series [34, 38, 73, 86, 88, 102, 118, 144, 159]. To fully standardize the pathologic diagnosis of HS, especially as it relates to differentiating LATE-NC + HS from other distinct disease processes (e.g., anoxic–ischemic episodes or seizures), will require additional work. Prior studies of HS-related histomorphology [45, 56, 160], and the recent work by Hokkanen et al. [47], provided methodologies to study HS pathologic features systematically.

In conclusion, despite challenges associated with a fast-moving research field, we found opportunities for recommendations to improve the precision of LATE-NC staging based on published reports and diagnostic experience. We emphasize that TDP-43 immunohistochemical assessment should be performed in all brain autopsies of older persons. The diagnosis of LATE-NC Stages requires sampling from three specific brain regions (Fig. 1, and Supplemental File 1, online resource). A specific rubric is presented in Table 1 and overall recommendations in Table 2. Recent literature indicates the scope of the challenge, even for this relatively simple pathologic staging system, as a wide variation of results was reported in the proportion of subjects with LATE-NC Stage 1 in a survey of community-based autopsy cohorts [113]. We hope that the present update will assist in efforts to increase standardization in the diagnosis of LATE-NC. As more observations are made across diverse autopsy cohorts, gathering detailed information on each case, including clinical, radiologic, laboratory, neuropathology, and genetic data, may facilitate future refinements of the classification and staging of LATE-NC.

**Table 2:**
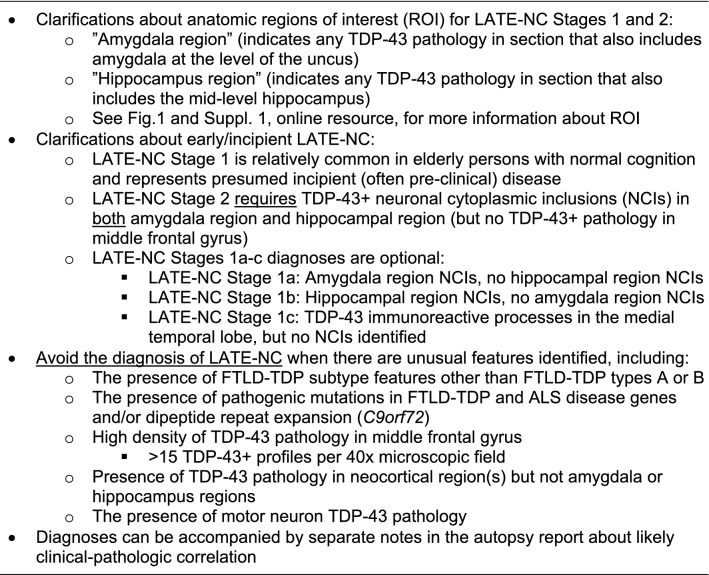
LATE-NC Stages based on anatomic distribution of TDP-43 pathology: updates and clarifications for routine autopsy diagnoses

## Supplementary Information

Below is the link to the electronic supplementary material.

Supplementary file1 (PDF 818 KB)

## Data Availability

As this is a review/consensus paper, there are no data to be made available.

## Abbreviations

ADNC: Alzheimer’s disease neuropathologic change
ALS: Amyotrophic lateral sclerosis
CTE: Chronic traumatic encephalopathy
FTD: Frontotemporal dementia
FTLD: Frontotemporal lobar degeneration
HS: Hippocampal sclerosis
LATE-NC: Limbic-predominant age-related TDP-43 encephalopathy neuropathologic change
NCI: Neuronal cytoplasmic inclusions
TDP-43: Transactive response (TAR) DNA-binding protein of 43 kDa
