# Supplementary material for: LATE-NC staging in routine neuropathologic diagnosis: an update
Source: Acta Neuropathol. 2022 Dec 13;145(2):159–73. doi: 10.1007/s00401-022-02524-2 (PMC9849315; doi:10.1007/s00401-022-02524-2)
Supplement: Supplementary file 1 — Supplementary file1 (PDF 818 KB) [file 401_2022_2524_MOESM1_ESM.pdf]

Supplemental File

Overview

As described in the update, there are 3 brain regions to be sampled for proper staging of LATE-NC. These 3 regions are the (a) Amygdala Region, (b) Hippocampus Region, and (c) Middle frontal gyrus. TDP-43 pathology in these sites correspond to LATE-NC Stages 1, 2, and 3, with optional subtypes for Stage 1 (see text for detail). Each of these regions is described further below with particular attention to the structures that comprise Amygdala (Stage 1) and Hippocampus (Stage 2) regions.

Amygdala Region

The recommended coronal section of amygdala is shown in the "Amygdala Region" panel shown below (an LFB/Nissl image with accompanying diagram of the same).

This coronal section is taken through the central portion of the Amygdala at the level of the uncus (*unc*). External landmarks in this region include the ambient gyrus (*AG*), overlying the junction of entorhinal cortex (*ERC*) and amygdala, the semilunar gyrus (*SLG*) overlying the amygdala proper, the anterior parahippocampal gyrus (*PHG*) containing *ERC* or Brodmann area 28 (*ERC* or BA 28), the rhinal sulus (*rs*), which is the anterior-most continuation of the collateral sulcus (*cs*), and the (lateral) occipitotemporal or fusiform gyrus (*OTG* or *FG*). In the medial bank of *rs* is the transentorhinal cortex (*TEC* or BA 35), also termed perirhinal cortex. Lateral to *rs* is temporal cortex, including Brodmann area 36 (BA 36) and neocortex of Brodmann area 20 (BA 20). ERC and TEC are part of the periallocortex that surrounds amygdala, and these may have isolated TDP-43-positive NCI pathology even without NCIs in amygdala proper (see text for detail).

The amygdala itself can broadly be divided into four regions, as shown here. These are the cortical-like region (*Co*), immediately deep to the pial surface of the *SLG*, the basal nuclei, divided into basomedial (*Bm*) and basolateral (*Bl*) nuclear groups, the lateral nuclear group (*Lat*) including its conspicuous, stripe-like lateral subdivision (*Lat-L*), and the dorsally located centromedian nuclear group (*Me/Ce).* The ventral extension of claustrum (*Cls*) and basal forebrain (not shown) are both closely associated with amygdala and are frequently sampled in this section. The

subamygdaloid white matter (*wm*) is a conspicuous site of TDP-43 positive "processes" in some patients (see text for detail).

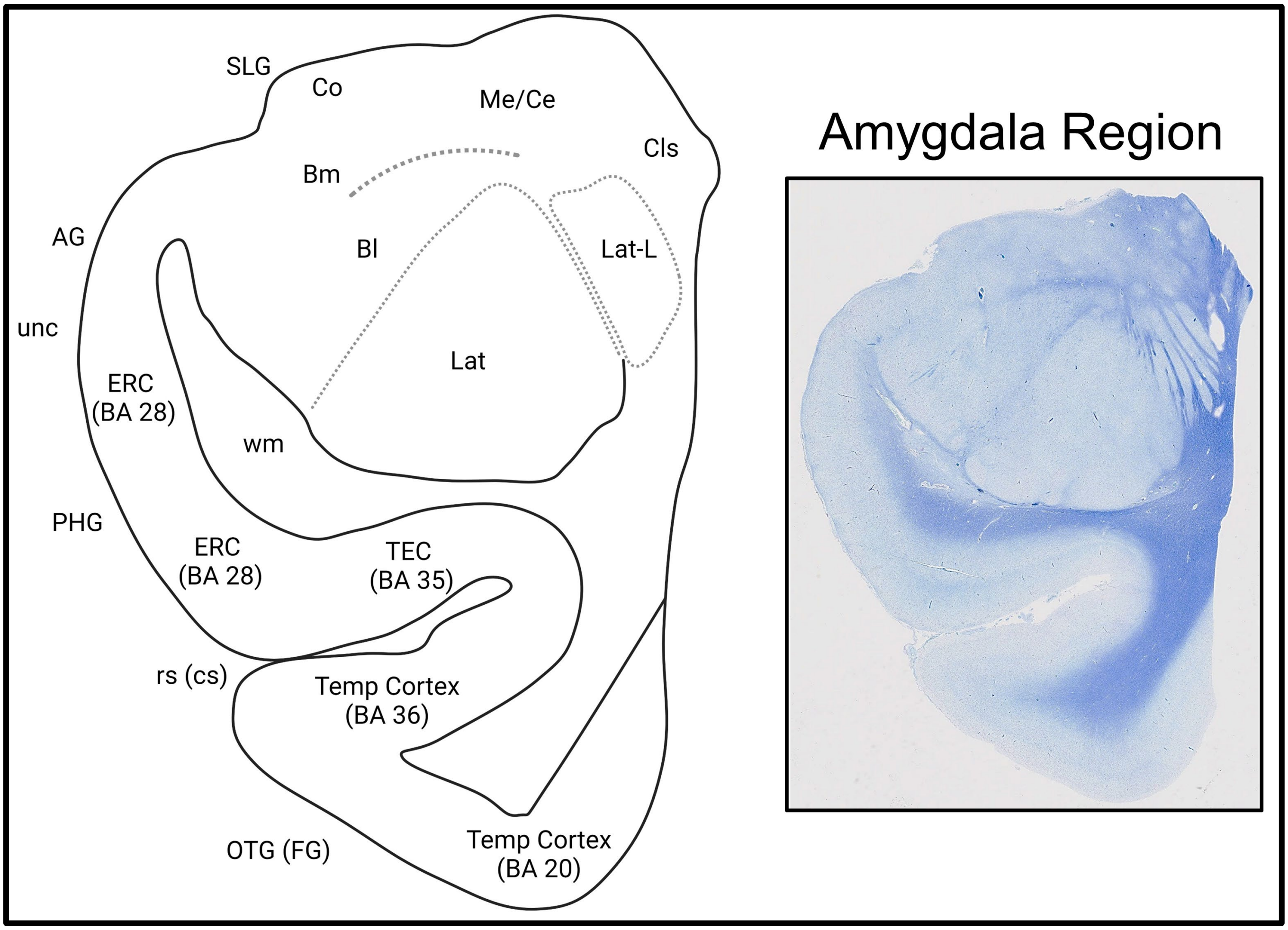

Though not shown here, sections taken at a slightly more posterior/ caudal level will demonstrate the temporal horn of the lateral ventricle. This portion of ventricle is variably dilated across patients, depending on the degree of limbic region atrophy. In addition, at these more posterior/ caudal levels, CA1 and subiculum of the anterior hippocampus begin to appear, positioned between amygdala dorsally and the ERC ventrally. As described in the text, in some samples, this anterior hippocampal region may have TDP-43 pathology in the absence of clear amygdala NCIs. As this pathology occurs in standard sections through uncus and amygdala, this pathology is also considered as being within "Amygdala Region" (see text for detail).

Hippocampus Region

The recommended coronal section of hippocampus is shown in the "Hippocampal Region" panel shown below (an LFB/PAS image with accompanying diagram of the same).

The recommended Hippocampus region section is taken at the level of the lateral geniculate nucleus (*LGN*). Dorsally located structures in this section also include medial geniculate nucleus (*MGN*), stria terminalis (*st*), tail of caudate nucleus (*Cd*) and tapetum (*tp*).

The hippocampal formation proper comprises the dentate gyrus (*DG*) and cornu ammonis. The granule cell layer of DG is a frequent site of TDP-43 NCI pathology in LATE-NC (dashed line). Components of cornu ammonis include sectors CA3, CA2, and CA1, as shown. Of these sectors, CA1 is a frequent site of neurite and NCI pathology in LATE-NC.

Medial to CA1 is the subiculum (*Sub*), the third region of the hippocampal formation. Like CA1 and DG, subiculum is a frequent site of TDP-43 pathology in LATE-NC. The border of CA1 and subiculum is ill-defined as these structures overlap (and their distinction is not relevant to staging LATE-NC). But at its more lateral extent, subiculum is recognized by being wider than CA1, by its greater neuron numbers, by slightly larger pyramidal cells, and at its

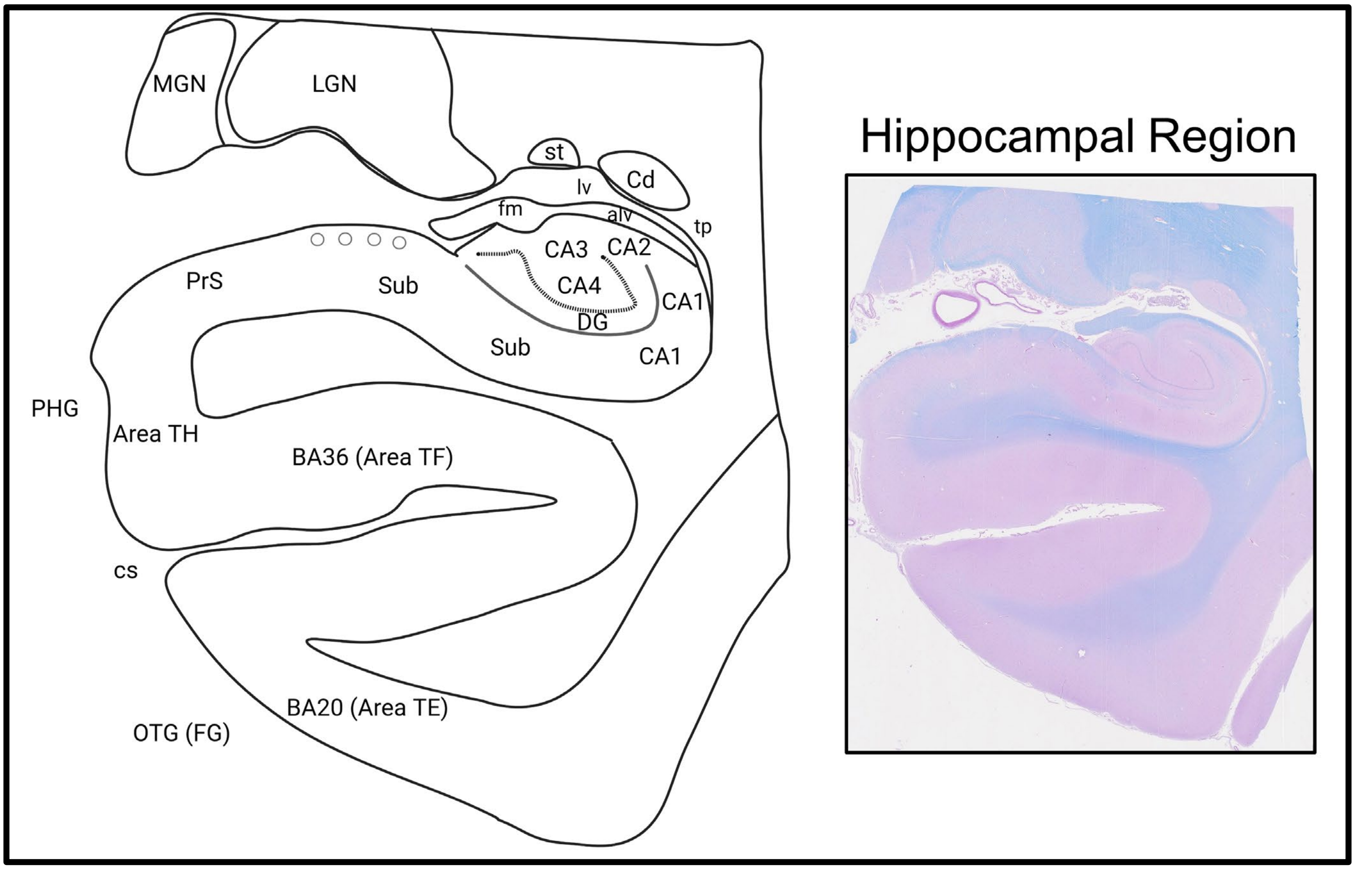

medial-most extent, by overlap with clusters of small neurons (indicated by small circles here) that are part of Presubiculum (*Prs*), a region that also extends medially onto the *PHG* surface.

Additional landmarks in this region include *cs*, and *OTG (FG*), as defined above under "Amygdala Region". Note that ERC (BA 28) is not present in this section. This portion of posterior parahippocampal cortex instead contains Von Economo areas TH and TF (BA 36). The fusiform gyrus lateral to *cs* contains neocortex of BA 20 (Von Economo area TE).

Middle frontal gyrus

For LATE-NC staging, cortex of middle frontal gyrus is ideally sampled at the level of the genu of corpus callosum and temporal poles. The gyrus is easily distinguished in this section between the superior and inferior frontal sulci (see manuscript Figure 1) and this neocortical region corresponds approximately to Brodmann area 46. Neocortical pathology in this region may be seen in stage 3 LATE-NC (see text for detail).

Note

Images created with BioRender.com. There is interindividual variability in sections through amygdala region and uncus, as well as in terminology applied in various references to both amygdala and hippocampal regions. As such, the labels applied above are based on consensus features in multiple references, in tandem with the features of the specific samples shown.

References:

(1) Amunts, K., Kedo, O., Kindler, M., Pieperhoff, P., Mohlberg, H., Shah, N. J., . . . Zilles, K. (2005). Cytoarchitectonic mapping of the human amygdala, hippocampal region and entorhinal cortex: intersubject variability and probability maps. Anat Embryol (Berl), 210(5-6), 343-352. doi:10.1007/s00429-005-0025-5

(2) Braak, H. (1980). Architectonics of the human telencephalic cortex. Berlin ; New York: Springer-Verlag.

(3) Braak, H., & Braak, E. (1991). Neuropathological stageing of Alzheimer-related changes. Acta neuropathologica, 82(4), 239-259.

(4) Carpenter, M. B. (1985). Core text of neuroanatomy (3rd ed.). Baltimore: Williams & Wilkins.
(5) Crosby, E. C., & Humphrey, T. (1941). Studies of the vertebrate telencephalon. II. The nuclear pattern of the anterior olfactory nucleus, tuberculum olfactorium and the amygdaloid complex in adult man. Journal of Comparative Neurology, 74(2), 309-352.
(6) Crosby, E. C., Humphrey, T., & Lauer, E. W. (1962). Correlative anatomy of the nervous system. New York: MacMillan.
(7) DeArmond, S. J., Fusco, M. M., & Dewey, M. M. (1989). Structure of the human brain : a photographic atlas (3rd ed.). New York: Oxford University Press.
(8) Economo, C., & Triarhou, L. C. (2009). Cellular structure of the human cerebral cortex (New English ed.). Basel ; New York: Karger.
(9) Mai, J., Majtanik, M., & Paxinos, G. (2016). Atlas of the Human Brain. Waltham, MA: Elsevier.
(10) Yilmazer-Hanke, D. M. (2012). Amygdala. In J. K. Mai & G. Paxinos (Eds.), The human nervous system (3rd ed., pp. pp. 759-835). Amsterdam ; Boston: Elsevier Academic Press.
